# Holistic perinatal care during intimate examinations: An integrative review

**DOI:** 10.4102/hsag.v30i0.3069

**Published:** 2025-12-19

**Authors:** Ntsoaki M. Tshabalala, Mariatha Yazbek, Carin Maree

**Affiliations:** 1 Department of Nursing Science, Faculty of Health Sciences, University of Pretoria, Pretoria, South Africa; 2 Department of Nursing Science, Faculty of Health Sciences, Sefako Makgatho Health Sciences University, Pretoria, South Africa

**Keywords:** guidelines, holistic, perinatal care, intimate examinations, Perinatal period

## Abstract

**Background:**

Intimate examinations are vital in midwifery, but without proper care and communication, they can cause distress, especially for first-time pregnant women. A holistic approach that addresses physical, emotional, social, psychological and spiritual needs is essential for ensuring comprehensive and compassionate care.

**Aim:**

To review existing literature on holistic perinatal care during intimate examinations.

**Method:**

An integrative review was conducted across relevant databases, which included Google Scholar, PubMed, Medline, Cumulative Index to Nursing and Allied Health Literature (CINAHL) and Elton Bryson Stephens Company (EBSCO) host to identify original research articles published in English between 2014 and 2024. In total, 67 articles which met the inclusion criteria were reviewed: 32 were international, 30 were from sub-Saharan Africa and 5 were from South Africa.

**Results:**

This study highlights two key aspects of holistic perinatal care during intimate examinations: *biological* and *psychosocial*. These aspects include health education, informed consent, decision-making involvement, emotional support, communication, counselling, social support, cultural competence and infection prevention to enhance women-centred care.

**Conclusion:**

Most studies focus on pregnancy-related interventions rather than holistic perinatal care during intimate examinations, and there are no maternity guidelines for such examinations in Africa. This review highlights the need for further research and outlines the biological, psychosocial and ethical considerations for improving perinatal care.

**Contribution:**

This study highlights the importance of holistic perinatal care during intimate examinations, emphasising biological, psychological and social aspects to enhance women’s experiences. It identifies gaps in maternity guidelines, particularly in Africa, and calls for further research to ensure comprehensive, women-centred care.

## Introduction

Intimate examinations, including breast, abdominal and vaginal assessments (Nelson 2021:23), are crucial in midwifery care but can be experienced differently by women, often leading to discomfort and stress (Zafra-Tanaka et al. 2019:4). This discomfort can be heightened for primigravida and women with a history of sexual abuse (Yildirim & Bilgin 2021:224), mainly when examinations are conducted insensitively or without adequate communication (Filej & Kaucic 2017:3; Quaresma et al. 2020:15). Many women prefer the presence of a chaperone during these procedures, which can help reduce embarrassment and discomfort. This preference is often influenced by the examiner’s gender (Murphy & Geary 2020:3). Holistic care, which addresses the physical, emotional, social, psychological and spiritual needs of pregnant women, is vital during the perinatal period women (Ventegodt & Ervin 2016:1938). Despite its importance, psychosocial issues such as antenatal and postnatal depression are frequently under-recognised and inadequately addressed, particularly when there is no continuity of care (Jahan et al. 2021:8). This review focuses on integrating holistic care into intimate examinations to enhance perinatal support and ensure that both physical and psychological aspects of maternal health are effectively managed (Lehman, David & Gruber 2017:10; Wade & Halligan 2017:998).

## Methodology of the integrative review

The integrative literature review aims to review various scientific literature sources about holistic perinatal care during intimate examinations. The methodology is discussed under the following steps of Souza, Da Silva and Da Cavharlo (2010), namely: preparing the guiding question, searching or sampling the literature, data collection, critical analysis of the studies, including the discussion of results and presentation of the integrative review (Souza et al. 2010:105).

### Step 1: Preparing the guiding question

The guiding question was prepared and defined to determine which studies need inclusion and the information gathered in each selected study (Souza et al.2010:105). The guiding question was: What is the existing literature or guidelines available for holistic perinatal care during intimate examinations? Although there are no guidelines or recent studies available on holistic perinatal care during intimate examinations, there have been several studies on the management of pregnant women during intimate examinations. Therefore, the focus was on holistic perinatal care during intimate examinations to fill an identified gap in perinatal care.

### Step 2: Searching or sampling the literature

A comprehensive search entailed using a predetermined period and keywords such as the title, abstract and the entire text screened for relevance by the authors about holistic perinatal care of women during intimate examinations.

An Internet search was implemented using Google Scholar, PubMed, Medline, CINAHL and EBSCOhost. These databases were utilised because they are the largest abstract and citation databases to provide superior support for the literature research process in academia and provide a global view to the researcher. Details included which published and unpublished literature were searched, how and by which mechanism they were searched, in addition to what the inclusion and exclusion criteria were. The researcher also checked if her study was not a duplicate. The following search terms were identified:

- Holistic perinatal care and intimate examinations.
- Maternity guidelines and holistic perinatal care.
- Maternity guidelines on intimate examinations.
- Midwives and holistic perinatal care.

The search results were initially broad but were subsequently narrowed by using specific search topics. The reference list from retrieved studies was manually searched. From 2014 to 2024, the reviewed literature comprises global research, including sub-Saharan Africa and South Africa. Using PubMed, 96 articles were found, 67 articles using CINAHL, 62 using Medline and 50 using Google Scholar, from which 275 articles and their abstracts were examined as to whether they met the inclusion criteria and addressed the research question.

#### Inclusion criteria

Articles published in English between 2014 and 2024 were utilised. The focus was on the holistic perinatal care and ethical manner aspects that needed consideration during intimate examinations addressed in current guidelines that include intimate examinations globally, including sub-Saharan Africa and South Africa. Articles published in peer-reviewed journals containing data on holistic perinatal care during intimate examinations were obtained. A total of 67 studies were obtained for full review.

#### Exclusion criteria

Articles already registered but unpublished were excluded from this study to reduce duplicate research and keep up-to-date integrative reviews. Letters, editorials and commentaries were excluded to increase the robustness of the study. Also excluded were grey literature and unavailable full-text articles and reviews. The researcher requested a librarian to review the literature for inclusion and exclusion criteria. Research on holistic perinatal care published in other languages was excluded from the study.

### Step 3: Data extraction and critical appraisal

**FIGURE 1:**
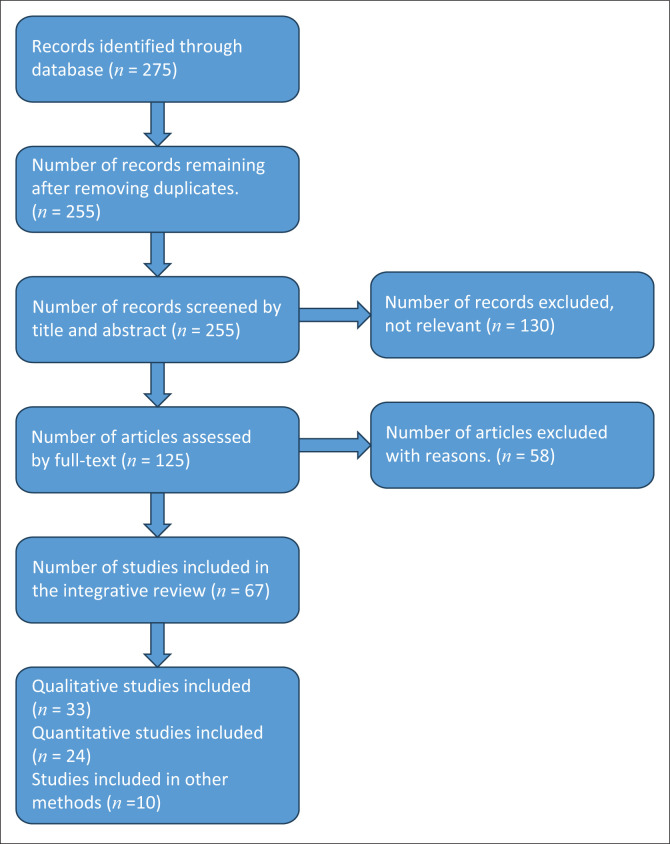
Preferred reporting items for systematic reviews and meta-analysis flow diagram for integrative review.

A PRISMA (Preferred Reporting Items for Systematic Reviews and Metanalysis) flow diagram (see Figure 1) was used to describe the inclusion process of the integrative review (Trifu et al. 2021:3). After an initial review of 275 studies, 20 duplicate studies were removed, leaving 255 studies that were retrieved and screened using inclusion and exclusion criteria, after which another 130 studies were discarded because of non-relevance. This left 125 primary studies meeting the inclusion criteria assessment of quality criteria. Another 58 studies were excluded as they did not meet the appraisal criteria. Furthermore, 20 studies focused only on maternal care during labour, 10 focused on factors contributing to a delay in labour, 12 focused on managing obstetric conditions and emergencies and 16 did not provide comprehensive data on holistic perinatal care.

A total of 67 studies were thoroughly reviewed, of which 33 were qualitative studies, 24 were quantitative studies and 10 other methods were retained and included in the integrative review because they provided data on holistic care of low-risk women during antepartum, intrapartum and postpartum during intimate examinations. Cross-sectional survey studies conducted on examination on the feeling of discomfort during vaginal examinations, lack of support, abandonment or neglect during pregnancy, history of abuse and sexual abuse and post-traumatic stress disorder in women to determine the correlation between these variables were also included. A prepared instrument was developed to extract data from selected articles to ensure the collection of all relevant data, minimise the risk of transcription errors, guarantee precision when checking the information and save it as a record. The final selected articles were based on authors, publication year, location, purpose, design and findings.

#### Study settings

Of the 67 articles reviewed, three were from the United States, 12 were from the United Kingdom, one was from England,five were from Australia, one was from Spain, one was from Iran, one was from Sweden, three were from India, four were from Turkey, three were from New Zealand, two were from Denmark, two were from Sri Lanka, two were from Congo, four were from Kenya, two were from Ethiopia, five were from Nigeria, two were from Zambia, one was from Switzerland, two were from Tanzania, one was from Denmark, three were from Israel, one was from Norway and five were from South Africa.

### Step 4: Critical analysis of the studies included

Following data extraction, a comparison was implemented across reports to interpret and synthesise the results, and 67 studies were thematically analysed based on the intervention options available to provide holistic care for pregnant, labouring and postnatal women during intimate examinations. Two main themes comprising eight subthemes were extracted from several studies and guidelines about holistic perinatal care (see Table 1).

**TABLE 1:** Themes and subthemes extracted from studies.

Themes	Subthemes
1. Biological factors	1.1. Increase understanding of physiological changes. 1.2. Minimise the risk of infection during intimate examinations.
2. Psychosocial factors	2.1. Strengthen psychological support. 2.2. Obtain informed consent. 2.3. Addressing individual unique needs. 2.4. Establish a midwife and patient relationship. 2.5. Social support during intimate examinations. 2.6. Consideration of cultural and religious factors that may affect the execution of intimate examinations.

The biopsychosocial model approach was used to discuss the results and themes systematically, considering psychosocial and biological factors and their complex interactions in understanding maternal health, complications, stressors and healthcare delivery (Wade & Halligan 2017:998).

Application of the biopsychosocial model to perinatal health benefits both maternal and neonatal health, given two decades of reliable evidence from large-scale studies linking perinatal maternal psychosocial well-being and neonatal physical health (Blount, McDonough & Gao 2021:9). Its application provides clues in improving maternal psychological well-being, particularly during intimate examinations (Taukeni 2019:3). This model criticised midwives by narrowing their focus to biomedical factors and to regard pregnant women as objects and for ignoring the possibility that the subjective experience of the pregnant women is amenable to scientific factors.

## Holistic perinatal care

### Biological factors

During the perinatal period, women face several biological implications that can hurt the women during intimate examinations (Ramiro-Cortijo et al. 2021:8). Biological factors such as physiological changes and infection need to be taken into consideration when executing the intimate examinations (Richardson et al. 2019:22). The following subthemes were identified from the studies, such as increased understanding of physiological changes and minimising the risk of infection during intimate examinations, thus reducing anxiety and making the experience of intimate examinations comfortable to the pregnant women.

### Increase understanding of physiological changes

Although pregnancy is a natural physiological event, it signifies significant changes in women’s lives, necessitating their adaptations to these changes (Janighorban, Kazemi & Haghani 2025:5). Physiological changes during the perinatal period significantly impact intimate examinations, such as hormonal changes, which can lead to increased vascularity and swelling of tissues, which may affect the examination experience. Studies by Kjerulff Madsen et al. (2019:12) indicated that changes in pain perception and increased sensitivity during pregnancy can affect how comfortable a patient feels during an intimate examination. Additionally, pregnancy-related physical changes can significantly influence a woman’s body image (Mukwege & Berg 2017:1446). Women’s views of their changing bodies during pregnancy vary, mainly depending on how they handle societal expectations of female beauty (Mukanga et al. 2021:1145). Hodgkinson, Smith and Wittkowski (2014:32) observed that these body changes often lead to discomfort or embarrassment, especially during intimate examinations where women must undress in front of midwives (Amaechina, Moodley & Ramnarain 2017:12). Furthermore, attending to women’s narratives about their pregnant bodies may identify at-risk women and provide an opportunity for health professionals to provide support to either address or accept body image dissatisfaction (O’Doherty et al. 2017:112). Clinical communication training may enable health professionals to explore body image concerns with women and guide them in identifying ways of accepting them (Bonnén et al. 2023:12).

Studies have shown that educating women during pregnancy about the physiological changes and hormonal changes can improve their adaptions to pregnancy and reduce stress, anxiety and discomfort during intimate examinations (Azene, Yeshita & Mekonnen 2019:116; Bonnén et al. 2023:14). Moreover, understanding the hormonal effects can guide practitioners in modifying their approach to avoid causing undue discomfort during intimate examinations (Wang et al. 2017:12). Employing gentle techniques, providing explanations and using pain management strategies can help alleviate discomfort (O’Doherty et al. 2017:23).

### Minimise the risk of infection during intimate examinations

Pregnant women have a high propensity to acquire the infections because of their altered physiological and immunological function (Omer, Ali & Babar 2020:247). Studies indicated that the risks of having infections during a vaginal examination are low if hygiene guidelines are correctly followed, and the clinical reasons for having one are clear (Pereboom et al. 2023:7). Midwives must maintain hygiene precautionary measures when executing vaginal examinations such as washing their hands, wearing sterile gloves, using lubricant and, if necessary, using sterile swabs and a speculum (Kumar, Saadaoui & Khodor 2022:5). Studies indicate that vaginal examinations may raise the risk of infections for both the mother and baby, and they can also heighten the likelihood of premature rupture of membranes (Omer et al. 2020:247). These are because of the increased vaginal discharge that commonly occurs during pregnancy and labour, which can complicate the examination and raise hygiene concerns (Kumar et al. 2022:5). Therefore, such examinations should only be performed when necessary. Several studies indicated that women experience many feelings such as embarrassment about undressing, worries about cleanliness, qualms about vaginal odour, concern that the midwives or obstetricians might discover something about sexual practices, fear of discovery of a pathological condition and fear of pain (Kourtis, Read & Jamieson 2014:68; Pereboom et al. 2023). Most of the abovementioned aspects may be even more conflicting when the gynaecologist is male (Omer et al. 2020:247). Women should be advised on good hygiene, and midwives should maintain infection control and refrain from keeping their nails long or putting long artificial nails to prevent unnecessary infection and harm to the women during intimate examinations (Kourtis et al. 2014:6).

### Psychosocial factors

Studies have shown that pregnancy is often viewed as a psychosocial event in and of itself, with several complex changes occurring and intimate examinations executed during the perinatal period (Dawson et al. 2020:709). Furthermore, the perinatal period, by its nature, is a stressful event, both influencing maternal and neonatal functioning (Adrine & Wayner 2019:3). Midwives and healthcare professionals should provide psychosocial support to women when executing intimate examinations to alleviate anxiety and fears (Dawson et al. 2020:709). The following subthemes were identified from the studies, such as strengthening psychological support during the perinatal period, obtaining informed verbal consent for intimate examinations, addressing women’s individual unique needs, maintaining patient and midwife relationships, support during intimate examinations and consideration of cultural and religious factors that may affect the execution of intimate examinations.

### Strengthen psychological support during the perinatal period

A holistic approach to health considers multidimensional elements of well-being, including psychological and emotional factors. According to the World Health Organization (WHO), psychological support of pregnant women during the perinatal period plays a vital role in their well-being (Mabetha et al. 2022:13). Pregnant women should be supported psychologically during intimate examinations to enhance their cooperation and alleviate their anxiety. Having good psychological wellness is an essential part of the perinatal period as it is associated with optimal perinatal outcomes (Sinha et al. 2019:49). While breast and vaginal examinations are common to gynaecological and obstetric encounters, vaginal examinations can be very challenging to the sexual abuse of survivors leading to perinatal depression and post-traumatic stress disorder (Lukasse et al. 2017:8). Studies have shown that to alleviate these feelings of discomfort, anxiety and depression, midwives and healthcare professionals must be supportive and create a conducive environment for this cohort (Watson 2017:13).

### Obtain informed verbal consent for the intimate examinations

Verbal consent is where a patient states their consent to a procedure verbally but does not sign any written form (Ghaedrahmati et al. 2017:5). The General Medical Council from Australia advises that it is necessary to maintain a professional boundary when examining women where intimate examinations may be involved, as they can be embarrassing and distressing to the women (Harrison et al. 2016:4). These examinations may have been perceived as threatening and sometimes intrusive and unconsented causing women with a history of sexual abuse to experience flashbacks to the traumatic abuse and dissociation (Roberts et al. 2021:7). Most of the studies found that the midwives should ensure that the patients receive sufficient information about the examinations to enable them to give informed consent (Nicholls 2021). Muliira, Seshan and Ramasubramaniam (2023:438) added that the procedures of intimate examinations should be explained to the pregnant woman in a language that the pregnant women understand. As supported by Gaedrahmati et al. (2017:5), women become more comfortable if sufficient information is given. Midwives should refrain from conducting intimate examinations in the presence of students or other persons without the patient’s consent (Nicholls 2021). Studies confirmed that women having procedures in intimate areas may generate a lot of anxiety and embarrassment, especially for those who are experiencing these procedures for the first time (Rivas 2017:11).

Midwives need to maintain their dignity and respect and build a sense of trust in the healthcare profession so that they do not feel ashamed to attend important antenatal care visits and maternity units (Walburg et al. 2014:21). Healthcare providers should explicitly ask permission to perform examinations and procedures and wait for consent before proceeding. They should not assume that a patient consents to a sensitive examination or treatment because they made an appointment (Harrison et al. 2016:5). Asking permission from the patient before the examination may help patients feel in control of their care, alleviate their anxieties and enhance their cooperation. Pregnant women should not feel that decisions regarding their perinatal are decided on their behalf, but they should be involved to make them feel in control (Murugesu et al. 2021:12). It may enhance their cooperation during procedures such as intimate examinations. According to Ebert et al. (2020:135), an explanation and requesting consent should be repeated before every subsequent intimate examination (Growe & Easten 2016:11).

### Addressing women’s individual unique needs

Woman-centred care prioritises the unique needs of each woman, focusing on her choice, control and continuity (Adu-Bonsaffoh et al. 2021:8). Studies suggest that allowing women to express their expectations and preferences can make intimate examinations more comfortable (Muliira et al. 2023:436). However, in Palestine, women reported feeling disrespected and lacking privacy during such examinations (Adu-Bonsaffoh et al. 2021:9), and verbal abuse during these procedures has been linked to post-traumatic stress disorder (Vazquez et al. 2021:9). Negative experiences can discourage women from further examinations, potentially leading to obstetric emergencies (Davies, Lund & Scheineder 2022:4).

Women with a history of abuse may experience heightened discomfort during examinations, exacerbated by unwelcoming environments and mistreatment (Guruge 2018:6; Mayra et al. 2022:9; Mkonyi, Mwakawanga & Rosser 2021:5). In Ireland, guidelines advocate for including abuse histories in antenatal records and addressing psychological issues discreetly (Pre-Hospital Emergency Care Council 2014:3). South Africa has introduced a screening tool for abuse and mental health, but its limited questioning may not capture sufficient information (Azene et al. 2019:11; National Integrated Maternal and Perinatal Care Guidelines for South Africa 2024:20). The 2019 South African Intrapartum Care Guidelines emphasise respectful, women-centred care but do not fully address the needs of women with psychosocial issues during intimate examinations (National Integrated Maternal and Perinatal Care Guidelines for South Africa 2024:16). Midwives often rush examinations, neglecting the emotional and psychological aspects, which can cause stress and reluctance in patients (Dabson et al. 2021:4).

Effective care requires midwives to be aware of trauma and prepared to respond to disclosures of abuse (Nerum et al. 2021:16), often necessitating an interprofessional team approach including social workers and mental health professionals (Faye 2020:27). The WHO (2018) recommends respectful maternity care, including effective communication and informed choice. Training midwives in woman-centred care can improve maternal outcomes (Mayra et al. 2022:12). Queensland guidelines also advocate for care that respects the dignity and considers psychological, social and cultural needs (Queensland Clinical Guidelines 2017:11). Increasing abuse in maternity settings highlights the need for counselling services and referrals to support abused women (Guruge 2018:16; United States Agency for United States Agency for International Development 2019:20; Wong et al. 2022:7). Midwives should individualise the approach to intimate examinations so that each woman’s sense of vulnerability, apprehension, fear and embarrassment are diminished to the extent possible (Lusambili 2020:13). A midwife’s job is not only to carry out a procedure or examination but to do so in a way that makes the patient feel reassured, comfortable and safe (Shamu et al. 2018:24).

### Establish midwife and patient relationships

Establishing a strong, supportive relationship between a woman and her midwife or maternity nurse is a crucial element of maternity care, as it significantly influences both the safety of the care provided and the woman’s overall experience (Mannava et al. 2021:12). Negative treatment, such as verbal abuse or physical aggression during intimate procedures, can severely impact a woman’s mental and physical health both immediately after childbirth and in the long term (Almorbaty et al. 2022:1328). Gaining insight into how these supportive connections are formed is essential, particularly given the numerous emotional, psychological and physical changes that women undergo during pregnancy and childbirth, all of which can affect maternal well-being and neonatal outcomes (Hoffmann et al. 2023:6).

Quality maternity care is grounded in the presence of a supportive relationship, built on mutual respect, shared decision-making and partnership between the woman and her healthcare provider (Almorbaty et al. 2022:1329). Effective communication plays a central role in fostering such relationships. According to Bradfield, Hauck and Duggan (2018:35), communication facilitates trust and connection, with midwives often employing strategies like building rapport and offering verbal support to nurture these bonds. Leinweber and Stramrood (2024:4) emphasised the importance of active listening as a core skill in creating supportive interactions, while Hoffmann et al. (2023:6) noted that insufficient interpersonal abilities among some midwives and maternity nurses often hinder effective communication, leading to women’s dissatisfaction and feelings of neglect.

### Social support during intimate examinations

Social support plays a crucial role in improving the physical and psychological health outcomes of mothers and childbirth companions (White 2021:4). The presence of a chaperone during birth and sensitive examinations enhances the overall birth experience and protects both patients and healthcare providers by serving as observers and witnesses (Mukanga et al. 2023:14; Price, Tracy & Upshur 2023:4). Chaperones can mitigate feelings of fear, vulnerability or embarrassment by providing psychosocial support and encouraging cooperation during intimate examinations (Campo 2018:34; Stanford et al. 2017:871).

The Association of Women’s Health, Obstetric and Neonatal Nurses (AWHONN) advocates for the patients’ rights to request chaperones during sensitive procedures (Price et al. 2015:4). Considerations such as age, cultural and religious beliefs, mental health, cognitive ability and history of sexual trauma may influence the decision to have a chaperone present (Price et al. 2023:5). Trauma-informed care should be a standard practice, as patients may not always disclose their trauma history (Tillman 2020). Chaperones should be trained, and policies should be in place to ensure their role and any concerns can be reported appropriately (Tavoli et al. 2016:5; Wanyenze, Maizne & Azna 2022:10).

Support groups for pregnant women and peer supporters can provide validation and comfort, particularly for marginalised or vulnerable mothers, complementing the work of health professionals (Nolan 2020:29; Rossman, Matine & Waonden 2022:10). Midwives should promote the importance of social support through their social circles, including the husband, mother, father and female networks during intimate examinations and ensure equitable treatment of women regardless of socioeconomic status (Stephenson, Shoeding & Wangtan 2018:14; Taylor 2020).

### Consideration of cultural and religious factors that may affect the execution of intimate examinations

According to Tavoli et al. (2016:5), cultural differences also have an impact. Hispanic women declared the utmost fear of embarrassment for a breast examination, followed by white and then black women. Studies found that 51.1% preferred to be examined by female healthcare professionals, 4.9% preferred to be examined by male healthcare professionals and 44% had no preference (Yanikkrem et al. 2019:506). Evidence shows that cultural and religious factors make nudity difficult and embarrassing to some women, which in turn makes intimate examinations difficult (David, Lund & Schneider 2018:24). For instance, inspection of the breasts requires a woman to undress to the waist and to sit upright with her arms behind her head. Moreover, some women found it difficult to expose their bodies and to allow them to be examined by male healthcare professionals because of cultural reasons (Tavoli et al. 2016:4).

Furthermore, studies supported that all sensible measures to reduce the extent and duration of nudity should be taken into consideration, which do not jeopardise the thoroughness of the examination, such as uncovering only one part of the body at a time (Bedaso et al. 2021:14; Yilmaz & Kucuk 2022:289), even during abdominal palpation. Another study stated that, instead of exposing the abdomen of the women, that midwives should adopt the use of the last regular menstrual period (LNMP) to estimate gestational age as it performs better than abdominal palpation (Nguyen et al. 2017:8). The Royal College of Obstetricians and Gynaecologists’ special practice guideline (2020:11) emphasises the importance of taking into consideration the cultural and religious aspects that can influence the execution of intimate examinations, where it highlighted that Hindu and Muslim women have strong cultural taboos against being touched by any man other than their husbands and have clear preferences for female doctors when such examinations are necessary.

It further describes the management of women who experience difficulty with intimate examinations, such as women with a history of sexual abuse and that they should be allowed to facilitate disclosure of any sexual abuse or trauma. None of these discussions should occur until the woman is fully dressed and is alone with the midwife or obstetrician (David et al. 2018:24).

## Discussion of results

An integrative review was done on holistic perinatal care during intimate examinations and confirmed that if pregnant women are provisioned with holistic perinatal care, their fears and anxiety will be reduced (Watson 2017:13). Cooperation of the women, especially primigravida, will be enhanced through holistic perinatal care, thus decreasing stress, postpartum depression and unnecessary referrals to tertiary maternity units (Alebel et al. 2018:10). Quantitative studies reviewed and confirmed that women having procedures in intimate areas may experience a great deal of anxiety, especially for those who are experiencing these procedures for the first time (Bonnén et al. 2023:12; O’Doherty 2017:112).

Some quantitative and qualitative studies indicated that women should be provided with holistic perinatal care during intimate examinations taking into consideration biological, psychological and social factors (Bonsaffoh et al. 2021:8; Nguyen & Latkin 2022:9; Nicholls 2021). Evidence suggests that cultural and religious factors can make nudity uncomfortable and embarrassing for some women, complicating intimate examinations (David et al. 2018:24). For instance, a breast examination requires a woman to undress to the waist and sit upright with her arms behind her head. Additionally, cultural reasons can make it difficult for some women to expose their bodies and allow male healthcare professionals to touch them (Tavoli et al. 2016:4). Midwives must consider women’s cultural and religious factors that may affect the execution of intimate examinations.

### Presentation of the integrative review

Application of the biopsychosocial model to provide holistic perinatal care can improve maternal and neonatal health outcomes. Studies confirmed that understanding and responding adequately to pregnant women’s stressors or suffering will improve their maternal health outcomes. Midwives must attend simultaneously to the biological, psychological and social factors to provide holistic perinatal care during intimate examinations (Blount et al. 2021:08).

During the perinatal period, women experience biological changes that can affect the comfort and safety of intimate examinations (Hodgkinson et al. 2014:7; Ramsay et al. 2016:14). These changes include hormonal shifts that increase tissue sensitivity and body image concerns. In order to enhance comfort and reduce anxiety during these examinations, it is crucial to understand these physiological changes and minimise infection risks. Pregnancy induces significant hormonal and physiological changes that affect a woman’s comfort during intimate examinations (Hunter, Hunter & Muncegn 2017:4). Educating women about these changes can help them adapt better and reduce discomfort (Dathe & Schafer 2022:788). Additionally, practitioners should be trained to handle these examinations gently and effectively, using pain management strategies as needed (Growe & Easten 2016:11).

Pregnant women are more susceptible to infections because of altered immune function (Pereboom et al. 2023:7). To prevent infections during vaginal examinations, hygiene practices such as hand washing, wearing sterile gloves and using appropriate equipment must be strictly followed (Kourtis et al. 2014:6). Examinations should be performed only when necessary to reduce infection risks and address women’s concerns about cleanliness and privacy.

The perinatal period can be stressful and emotionally taxing, influencing both maternal and neonatal health anxiety (Mabetha et al. 2022: 13). Providing psychosocial support and respecting individual needs during intimate examinations is essential for reducing anxiety and improving the overall experience (Bedaso et al. 2021:13). Providing psychological support during perinatal care is crucial for enhancing women’s cooperation and reducing anxiety. This support is especially important or vital for those with a history of trauma, as it can alleviate feelings of discomfort and depression associated with intimate examinations.

Ensuring that women understand and consent to intimate examinations verbally is critical. Clear communication about the procedures helps alleviate anxiety and maintains patient dignity (Nerum et al. 2021:16). Consent should be explicitly obtained before each examination, and procedures should be explained in an understandable manner.

Woman-centred care involves recognising and addressing each woman’s unique needs and preferences (Bradfield et al. 2018:35). Respecting personal histories and preferences can improve comfort and cooperation during intimate examinations, particularly for women with past trauma or abuse experiences (Mannava et al. 2015:12). Building supportive and respectful relationships between midwives and patients is key to quality maternity care (Schroll, Kjaergaad & Midtgaad 2017:5). Effective communication and mutual respect contribute to positive outcomes and enhance the examination experience (Mayra et al. 2022:17).

The presence of a supportive person, such as a chaperone, during intimate procedures can reduce feelings of vulnerability and embarrassment (Mukanga et al. 2023:14; Stephenson et al. 2018:14). Social support and peer networks play a significant role in improving maternal experiences and outcomes (McLeish & Redshaw 2015:11; Nolan 2020:9). Cultural and religious beliefs can significantly impact how intimate examinations are perceived and conducted (David et al. 2018:24; Nguyen et al. 2017:8).

Practitioners should be sensitive to these factors, minimising nudity and respecting preferences regarding gender and examination practices to accommodate diverse needs and beliefs (Omer et al. 2020:247).

## Conclusion

Concerning the integrative review of literature, maternity guidelines on intimate examinations to provide holistic perinatal care during intimate examinations were evaluated and came forward with existing evidence on holistic perinatal care during intimate examinations. The focus was on the biological, psychosocial factors and ethical aspects that need to be considered during intimate examinations addressed in the existing literature and current guidelines that include intimate examinations globally, in sub-Saharan Africa and South Africa. Most of the retrieved studies focused on interventions during pregnancy and not on holistic perinatal care during intimate examinations. It is worth noting that no maternity guidelines for intimate examinations were available in Africa. Hence, holistic perinatal care during intimate examinations in Africa needs further research to ensure the provision of quality care. The strength of the review lies in the concise summary of the biological and psychosocial aspects and ethical manner that need to be considered by midwives and healthcare professionals during intimate examinations. The results will be used to develop guidelines for holistic perinatal care during intimate examinations.
